# Tuberculous Disease of the Hips.—II

**Published:** 1909-03-20

**Authors:** 


### Surgery.

### TUBERCULOUS DISEASE OF THE HIPS.—II.

In the previous article the various stages of this affection were described which occur previous to abscess formation; and by abscess in this connection we mean a collection of pus which makes its way to the surface and becomes evident in some superficial position. It is not really correct to use the word in this restricted sense, since as soon as dorsal dislocation makes its appearance it is probable that there is already pus in the joint, and therefore that there is, truly speaking, an abscess formed. The pus may make its way to the surface at almost any point, but the common sites at which it presents are (1) on either side of the tensor fasciæ femoris; (2) in Scarpa's triangle; (3) in the gluteal region; and (4) above Poupart's ligament. When pus appears in the last situation it has almost certainly made its way through the acetabulum and so into the pelvis; for retro-peritoneal collections of pus in the pelvis have a habit of pointing just above Poupart's ligament. This happens also in a psoas abscess. More rarely the pus may spread from the joint into the bursa under the tendon of the psoas and invade the psoas muscle itself, causing a fluctuating swelling above and below Poupart's ligament—fluctuation being obtained from one part of it to the other. Another position in which abscess formation may develop is the chain of inguinal lymphatic glands. Apart from disease of the hip-joint tuberculous disease of the inguinal glands is not very uncommon, and in all cases where these glands are enlarged without an obvious primary focus being discovered this should be borne in mind. It is a fact on which due stress is not laid in text-books of surgery. To return, however, the fact that the abscess occasionally makes its way into the pelvis is important; it is easily overlooked unless a rectal examination is made in all advanced cases. An occasional complication of the later stages is the onset of amyloid disease. To those who have any clinical experience of this condition the appearance of the patient is in itself highly suggestive. The skin appears yellow and almost transparent, and the veins are more than normally apparent. The urine, however, should be examined; it is increased in quantity and contains much albumen, but its specific gravity is low.

The degree of success attending the treatment of a tuberculous hip depends upon a number of factors, but the chief of these are the commencement of treatment in an early stage and the perseverance with which it is carried out by both the patient and the medical man. Bad results are more commonly seen among the poorer class of patients than among the well-to-do, because the treatment involves keeping the patient completely at rest for a considerable period.

As was said in the previous article, the disease is rarely diagnosed before some flexion of the joint has occurred. Attention must therefore be directed in the first place to getting the limb straight. This is best effected by means of an ordinary extension apparatus. The contraction is due to muscular spasm only, and nothing will overcome this so well as the constant pull of a weight suspended from a pulley. The amount of weight necessary varies in each individual case, but a good general rule is to allow half a pound for each year of the patient's age. The flexion will usually be completely overcome in a few days; it hardly ever takes longer than a week or two in the early stages.

The best method of telling whether the leg is straight is to flex the sound limb well on to the abdomen; if the affected limb can be kept straight *without lordosis* the extension has done its work, and now an apparatus should be applied to immobilise the joint. Nothing has so far been invented to answer this purpose better than a Thomas' hip splint. It is usual to leave the fitting of this to an instrument-maker, but the plan is not a sound one. Every medical man should be able to measure a patient for a Thomas' hip splint, and to tell whether the apparatus fits satisfactorily or not.

The splint consists of a flat bar of malleable metal which is applied to the back from the scapula to below the knee; this is shaped to fit the curves of the body, and is fixed by three horizontal pieces which are fastened to it behind and surround respectively the body at the level of the nipples, the thigh, and the calf. The splint thus prevents all movement at the joint. A patten is placed on the sole of the boot of the sound leg, and the patient can get about with the aid of crutches. He should wear this for a considerable period—*i.e.* for at least six months after all active signs of disease have disappeared. When he leaves it off he should still use the crutches until he gradually accustoms himself to using the limb again. Curiously enough, ankylosis of the joint due to adhesions does not often seem to follow.

Several accessory methods of treating tuberculous joints, apart from fresh air and good hygiene, have recently been advocated. One of these is Von Bier's method of inducing an artificial hyperæmia by means of an elastic tourniquet applied to the limb. In the case of the hip, however, the method is not as easily applicable as in other joints because of the practical difficulty of constricting the limb at this high level. The results of the treatment do not seem uniformly satisfactory.

The other method to which we refer is the injection of tuberculin T.R. Very successful results are reported from its use, but it is difficult to estimate in any given case how much of the improvement is due to the tuberculin and how much to enforced rest. We do believe, however, that tuberculin has a definite therapeutic value, and that it hastens the clearing up of active signs. But it should only be used in conjunction with enforced rest; under no circumstances should it be substituted for it. Rest is the only treatment of any real value, a fact which was pointed out many years ago by Hilton, and his words are as true to-day as they were when he wrote them. The operative treatment of the later stages will be considered in a succeeding article.

